# Osteoarticular tuberculosis of the ankle, a rare localization: a case report

**DOI:** 10.1099/acmi.0.000654.v3

**Published:** 2023-10-25

**Authors:** Zakaria Malihy, Elmostafa Benaissa, Yassine Ben Lahlou, Adil Maleb, Mostafa Elouennass

**Affiliations:** ^1^​ Department of Bacteriology, Mohammed V Military Teaching Hospital/Faculty of Medicine and Pharmacy (University Mohammed V), Rabat, Morocco; ^2^​ Laboratory of Microbiology, Mohammed VI University Hospital/Faculty of Medicine and Pharmacy (University Mohammed the first), Oujda, Morocco

**Keywords:** ankle, PCR, tuberculosis

## Abstract

Tuberculosis poses a considerable public health problem in countries where the disease is endemic. Osteoarticular tuberculosis represents 3–5 % of all tuberculosis cases and 10–15 % of extra-pulmonary tuberculosis cases. Involvement of the foot and ankle is rarer. We report a case of osteoarticular tuberculosis of the ankle in a 71-year-old patient with type 2 diabetes and hypertension who presented to the trauma department of the Mohammed V Military Hospital with a painful swelling of the ankle. Standard X-rays and computed tomography scans of the ankle showed inflammatory involvement of the bone and joints. Antitubercular therapy was instituted. Given the context of endemicity, any atypical presentation of lingering bone lesions should raise the suspicion of an osteoarticular tuberculosis in order to ensure early therapeutic management.

## Data Summary

No data were reused or generated in this study.

## Introduction

Tuberculosis represents a significant public health problem and ranks among the top 10 causes of death worldwide. According to the World Health Organization (WHO), Morocco reported nearly 35 000 cases of tuberculosis in 2021.

Osteoarticular tuberculosis accounts for a relatively small percentage of all tuberculosis cases, approximately 3–5 %, and a larger proportion, around 10–15 %, of extrapulmonary tuberculosis cases [1, 2]. Among the osteoarticular manifestations, tuberculous spondylodiscitis is the most common, accounting for approximately 50 % of cases, followed by tuberculous arthritis and extra-vertebral tuberculous osteomyelitis [3]. In contrast, involvement of the foot and ankle is rarer [4, 5].

In this report, we present a rare case of osteoarticular tuberculosis affecting the ankle of a 71-year-old patient with diabetes and hypertension. We also underline the importance of molecular biology techniques in the early diagnosis and management of such uncommon pathologies.

## Case report

The case involved a 71-year-old patient with a medical history of type 2 diabetes, hypertension and right hemiparesis due to a prior ischaemic stroke, without any surgical or allergic history, and no known exposure to tuberculosis. The patient was admitted with swelling in the right ankle that had been present for over a month, with no history of trauma. The patient did not report any signs of infection, such as cough, burning during urination or diarrhoea, nor signs of tuberculosis infection such as fatigue, fever or night sweats.

Clinical examination revealed an afebrile patient with normal conjunctivae. At the ankle level, there was warm, red, fluctuant and painful swelling. Joint mobilization was painful.

Neurologically, the patient was conscious (Glasgow Coma Scale score of 15/15), well-oriented in time and space, and did not exhibit any sensory or motor deficits. Pupils were equal and reactive.

On respiratory examination, the respiratory rate was 18 cycles per minute, with no cyanosis, digital clubbing or thoracic deformities observed. There were no signs of respiratory distress or paradoxical breathing. Breath sounds on lung and chest examination were clear, and oxygen saturation was at 94 %.

The patient was haemodynamically stable with a blood pressure of 120/50 mmHg, showing no signs of hypoperfusion, and had a regular and strong pulse with a heart rate of 74 beats per minute. Regarding cardiac auscultation, both the first heart sound (S1) and the second heart sound (S2) were clearly audible.

On a biological level, the complete blood count (CBC) showed lymphopenia at 0.5 G l^−1^ and normochromic microcytic anaemia at 9.5 g dl^−1^, while the remaining CBC data showed no abnormalities. The peripheral blood smear was normal with no atypical cells. Fibrinogen level was at 9.1 g l^−1^. The activated partial thromboplastin time (APTT) ratio and prothrombin time (PT) were 1.6 and 61 %, respectively.

On renal examination, urea and creatinine levels were normal at 0.25 g l^−1^ and 6 mg l^−1^, respectively. C-reactive protein (CRP) was 138.7 mg l^−1^, and total proteins were 58 mg l^−1^. No hydro-electrolytic abnormalities were found in the blood electrolyte panel. Liver function was normal, with aspartate aminotransferase (ASAT) at 8 IU l^−1^ and alanine aminotransferase (ALAT) at 12 IU l^−1^. Serology for hepatitis B virus (HBV), hepatitis C virus (HCV) and human immunodeficiency virus (HIV) were all negative.

On day 1 of admission, the patient underwent joint drainage with a lavage procedure for abscess evacuation, during which pus and joint lavage fluid were collected for cytobacteriological analysis. Direct examination of the deep pus and lavage fluid, stained by Gram’s method, revealed an inflammatory cellular response primarily consisting of polynuclear leukocytes with a more significant presence in the pus. No bacterial flora was detected.

The samples were cultured on Columbia agar with 5 % sheep blood, on Polyvitex chocolate agar and in Brain-Heart Infusion (BHI) broth for enrichment, which was then subcultured onto blood agar. Incubation was performed aerobically at 37 °C for 18–24 h. Schaedler agar and Columbia blood agar supplemented with nalidixic acid and colistin were inoculated and incubated anaerobically at 37 °C for 48 h. Anaerobic conditions were achieved using an anaerobic jar (Oxoid) and an anaerobic gas-generating system (Anaerogen; Oxoid). Observation of the culture plate was done every 24 h for cultures incubated aerobically and every 48 h for cultures incubated anaerobically. After 15 days of incubation, all cultures and subcultures returned as sterile.

On day 21, due to the lack of clinical improvement, the patient underwent a second procedure for joint drainage and lavage. Due to the persistent sterility of the cultures and the chronic nature of the lesion, the pus and joint washing fluid were sent to the microbiology lab where a search for mycobacteria was conducted in addition to the standard cytobacteriological examination to search for common pathogens. For the latter, the culture media used were the same as previously mentioned and were incubated and examined in the same manner. All of the media returned sterile results.

**Fig. 1.**
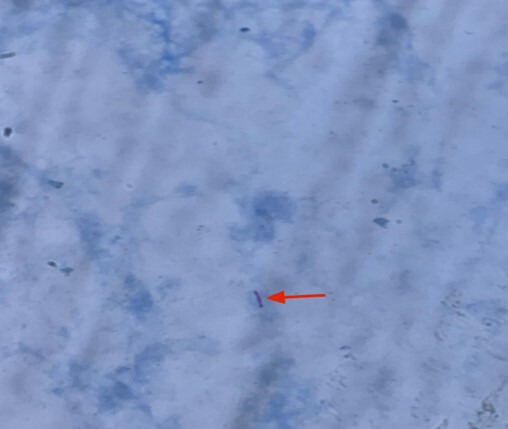
An acid-fast bacillus (AFB; red arrow) found on direct examination using Ziehl–Neelsen staining (Magnification 1000x).

Mycobacterial testing was conducted on the lavage fluid and revealed acid-fast bacilli (AFB) on direct examination (Fig. 1) using the Ziehl–Neelsen staining method (1–10 AFB per 100 fields). Cultures on solid Löwenstein–Jensen medium and in liquid MGIT (Mycobacteria Growth Indicator Tube) medium were positive at day 21 and day 10, respectively. Real-time PCR (GeneXpert MTB/RIF; Cepheid) detected the *Mycobacterium tuberculosis* complex at very low levels with no detection of rifampicin resistance.

Soft tissue ultrasound revealed a poorly defined hypoechoic, heterogeneous swelling that extended into the joint, with infiltration of the muscular and tendinous structures. This suggested a complicated abscessed tenosynovitis and myositis.

**Fig. 2.**
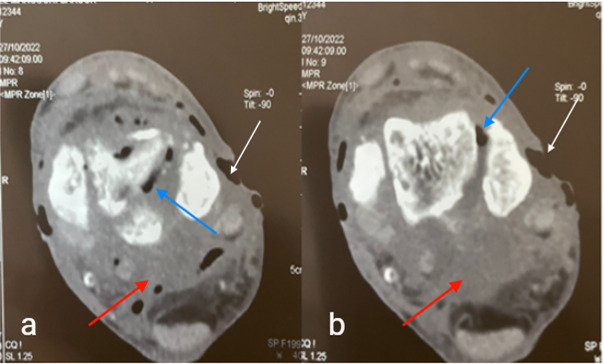
Axial ankle CT (a and b) scan showing diffuse infiltration of the soft tissues (red arrows) with significant gas extending from subcutaneous soft tissues and penetrating between bone structures (blue arrows). Note loss of cutaneous substance (white arrows).

Computed tomography (CT) scan of the ankle showed diffuse infiltration of the soft tissues with significant gas extending from subcutaneous soft tissues and penetrating between bone structures (Fig. 2).

**Fig. 3.**
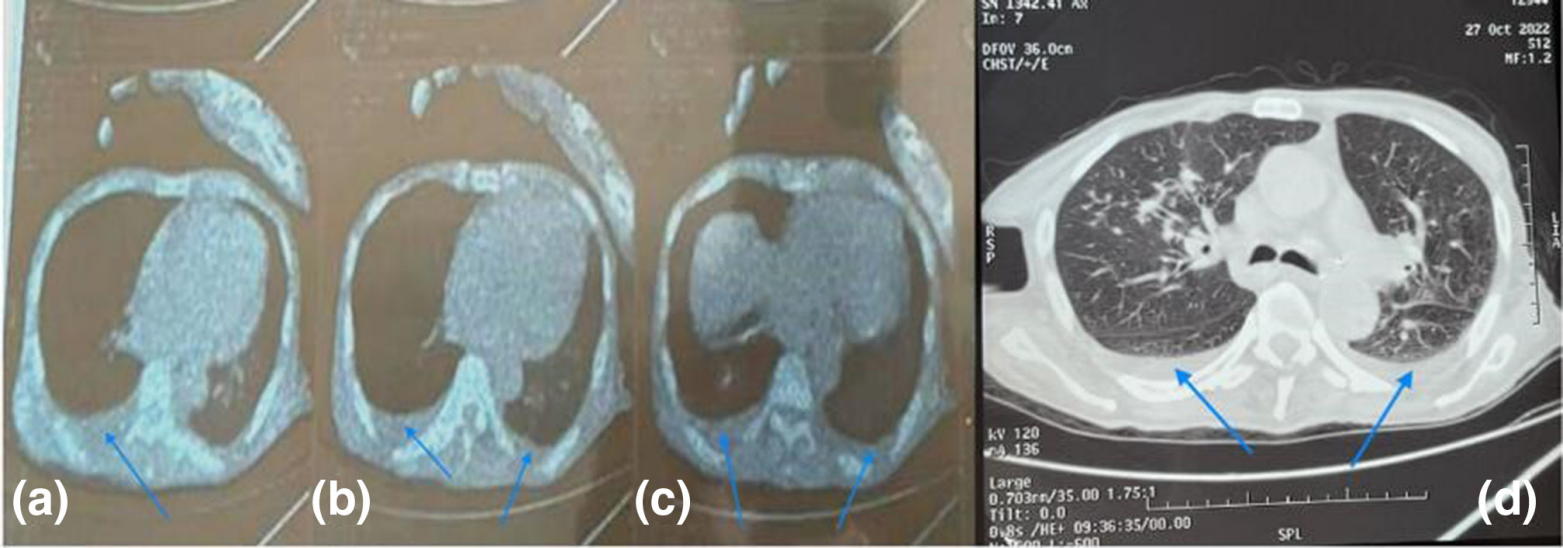
Axial CT scan mediastinal (a, b and c) and lung (d) windows showing bilateral pleural effusion (blue arrows) which is more pronounced on the right side.

A thoracic CT scan, conducted at the request of the pulmonologist to investigate the extent of the disease, revealed a bilateral pleural effusion which was notably more pronounced on the right side (Fig. 3). The limited amount of pleural fluid made it impossible to perform a puncture.

Following detection of the *M. tuberculosis* complex, a multidisciplinary collaboration involving a pulmonologist, traumatologist and microbiologist took place to discuss treatment. The therapeutic decision was to initiate anti-tuberculosis treatment in accordance with Morocco’s national tuberculosis control programme. The treatment was successful, resulting in significant clinical, biological and radiological improvement.

## Discussion

Tuberculous osteoarthritis typically arises from haematogenous dissemination originating from an initial infection, often in the lungs, lymph nodes or another organ. This primary infection can be either symptomatic or asymptomatic [5]. Osteoarticular tuberculosis predominantly manifests in immunocompromised individuals, such as those with HIV infection, undergoing corticosteroid therapy, receiving immunosuppressive treatments, having diabetes or with chronic renal failure. This condition exhibits a bimodal age distribution, with a peak occurrence at around 55 years in native populations and another peak between 20 and 35 years among immigrants [6]. The least common location is the ankle, accounting for less than 1–6 % of cases [7].

The atypical location, subtle clinical presentation, initial absence of a diagnosis of pulmonary tuberculosis and the presence of hemiparesis collectively contributed to the delayed diagnosis in this case. Typical tuberculosis impregnation signs such as night sweats and general deterioration are infrequent. Instead, the primary clinical manifestations are pain, swelling and functional impairment [8]. The chronic nature suggests a probable tuberculous aetiology.

Chest X-ray and CT scans lack specificity in such cases. In fact more than 80 % of patients do not have concomitant active tuberculosis [9, 10]. Standard ankle radiography and ankle CT scans are also non-specific [9, 10]. However, these examinations are valuable in detecting lesions and assisting in determining their nature. Magnetic resonance imaging is the preferred imaging examination as it offers the advantage of early detection, starting from the initial stages of infection, allowing for visualization of affected bone structures and their extension into surrounding soft tissues and adjacent joints [8].

In our case, the appearance of the thoracic CT scan was suggestive of pleurisy, raising the suspicion of a tuberculous origin. An anatomopathological study, while specific, was not conducted due to the purulent nature of the sample.

Laboratory tests revealed the presence of an inflammatory syndrome. Elevated CRP and fibrinogen levels and a significant cellular reaction in the pus and joint washing fluid, coupled with sterile cultures, prompted the investigation for Koch’s bacillus using molecular methods and as well as conventional classical methods. In cases of extrapulmonary tuberculosis, samples often contain few bacteria (paucibacillary), highlighting the importance of molecular methods. These methods offer high sensitivity and specificity (92–98 %) for diagnosing the *M. tuberculosis* complex, provide rapid results (within 2 h) and exhibit a strong positive predictive value for detection of rifampin resistance (98 %) [11]. Direct examination and culture remain essential components of the diagnostic process. Culture on solid media provides strains for in-depth studies, particularly in cases of therapeutic failure where exploring drug resistance is crucial. Additionally, culture in liquid media compensates for the slow growth observed on solid media. A positive direct examination confirms the presence of *Mycobacterium* spp. by visualizing AFB.

Treatment depends primarily on anti-bacillary drugs, which serve to inhibit the progression toward potential sequelae, such as chronic pain and deformity [8]. In most cases, lesions tend to heal within 6–12 weeks with appropriate medical treatment [12].

Surgical treatment is recommended when medical treatment fails, and when there are persistent issues such as synovitis, fistulas or abscesses [8]. With the introduction of four-drug anti-tuberculosis chemotherapy, surgical indications have become highly limited and selective. They are focused primarily on preventing or correcting deformities and enhancing the function of the affected joint [12]. Arthrodesis procedures are primarily recommended for the foot and ankle [8].

## Conclusion

In an endemic context, any unusual presentation of persistent bone lesions should prompt a suspicion of osteoarticular tuberculosis to ensure timely therapeutic intervention. The management of osteoarticular tuberculosis is multidisciplinary and necessitates coordination among physicians, bacteriologists and surgeons.
